# Expression of CD44, Transforming Growth Factor-β, and Matrix Metalloproteinases in Women With Pelvic Organ Prolapse

**DOI:** 10.3389/fsurg.2022.902871

**Published:** 2022-07-15

**Authors:** Weiwei Ying, Yanping Hu, Haibin Zhu

**Affiliations:** ^1^ Department of Gynecology, Taizhou Hospital of Zhejiang Province, Zhejiang University, Taizhou, China; ^2^ Department of Gynecology, The First Affiliated Hospital, Zhejiang University School of Medicine, HangZhou, China

**Keywords:** CD44, extracellular matrix, matrix metalloproteinase, pelvic organ prolapse, transforming growth factor-β

## Abstract

**Background:**

Defects in the pelvic floor connective tissue may underlie the etiology of pelvic organ prolapse (POP). We hypothesized that the expression of proteins regulating extracellular matrix turnover is altered in the uterosacral ligament of women with POP. We compared the expression of CD44, transforming growth factor (TGF)-β, and matrix metalloproteinases (MMPs) 2/9 in women with and without POP.

**Methods and Results:**

This matched case-control study included 30 postmenopausal women, with POP stage 2 and higher according to the POP quantification system, and 30 postmenopausal women without POP. Immunohistochemical analyses of the uterosacral ligament specimens obtained after hysterectomy were performed to determine CD44, TGF-β, MMP-2, and MMP-9 expression. The expression was quantified using ImageJ software, and the association between prolapse occurrence and risk factors was evaluated using Spearman's correlation analysis. CD44 expressions were significantly lower (*p* < 0.05), whereas MMP-2 and MMP-9 expression was higher (*p* < 0.0001 and *p* < 0.05, respectively), in the POP group than in the control group. The expression of TGF-β was similar in both groups. The occurrence of uterine prolapse was positively correlated with age, postmenopausal age, and MMP-2 and MMP-9 expression (*p* < 0.01) and negatively correlated with CD44 expression (*p* < 0.05).

**Conclusion:**

CD44, MMP-2, and MMP-9 may play critical roles in the pathogenesis of POP and may be candidate biomarkers of POP progression.

## Introduction

Pelvic organ prolapse (POP) is a birth-induced joint urinary/gynecological condition that affects one in four women of all ages and more than 50% of parous women over the age of 50 (1). POP is the most prevalent among women aged 60–69 years (2). The incidence of POP is 3%–6% based on symptoms, and can reach 50% based on vaginal examination; among patients with symptoms, 6%–18% receive surgical treatment. The requirement for medical assistance to treat pelvic floor defects is growing twice as fast as the population (3). Therefore, the prevention of POP has become an important issue in obstetrics and gynecology.

Pelvic organs are supported by the pelvic floor muscle complex, main ligament, uterosacral ligament (USL), and pelvic fascia. The pelvic fascia is a connective tissue structure that covers the walls and floor of the pelvis. It consists mostly of collagen fibers interlaced with elastin, smooth muscle cells, and fibroblasts, is abundant in extracellular matrix (ECM) that envelops the USL-cardinal ligament, and is thought to provide a critical level of support. The USL is one of the major ligaments holding the uterus and, thus, is a very important component of the pelvic support system. The mechanisms underlying pelvic floor laxity are not clearly understood. Studies in humans and animals have suggested that USL repair defects are due to changes in the structure and organization of fibrous connective tissue (4), including the amount and composition of the ECM, which is a major factor affecting tissue stability (5).

Collagen and elastin are the primary constituents of the ECM that provide strength and flexibility to the USL. A decrease in collagen deposition or changes in the proportion of collagen types can affect tissue stiffness and increase the susceptibility to POP. Type I and type III collagens are responsible for the stiffness and elasticity of connective tissue, respectively (6). Collagen is degraded by enzymes of the matrix metalloproteinase (MMP) family, which have distinct activities and functions. Among the MMPs, MMP-2 and MMP-9 break down degenerated collagen and elastin peptides into smaller fragments and are important factors in ECM turnover (7).

Transforming growth factor (TGF)-β is a fibrogenic cytokine that plays a key role in ECM remodeling and regulation of tissue integrity. TGF-β activity can alter the balance between ECM synthesis and degradation and is implicated in the pathophysiology of many fibrotic disorders, including pulmonary fibrosis (8) and cardiac fibrosis associated with heart failure (9). Furthermore, some studies show that TGF-β expression is altered in fibroblasts and pubic fascia of women with POP (10, 11). However, some reports show that TGF-β is not associated with the prognosis for patients with POP. Thus, there is no consensus regarding the role of TGF-β in the pathogenesis of POP, and further investigation is required.

The transmembrane glycoprotein CD44 is a major cell surface receptor for hyaluronic acid and can also interact with other ECM components, such as collagen and laminin. CD44 is implicated in the regulation of numerous attachment-dependent processes, such as cell proliferation, migration, and differentiation (12). Furthermore, extensive evidence indicates that CD44 plays a role in fibroblast invasion and progressive fibrosis of the lung and heart, not only through the establishment of specific transmembrane complexes but also through its participation in key signal transduction cascades (13–16). These data suggest that CD44 may be incorporated into the biochemical pathways responsible for the pathogenic alterations in the ECM that may promote POP. However, the role of CD44 in POP has not been investigated and, to the best of our knowledge, there have been no studies on CD44 expression in the USL of women with POP.

The main objective of this study was to compare the expression levels of CD44, TGF-β, MMP-2, and MMP-9 in the USL of patients with and without POP. We hypothesized that CD44 would be lower in the tissues of prolapse patients compared with those of the control group. We believe that this is the first report to illustrate the difference in CD44 expression between POP and non-POP women, aa well as the correlation between these indicators and uterine prolapse.

## Materials and Methods

Clinical information, including follow-up data, was extracted from the Department of Gynecology database between 2020 and 2021. Ethical approval was obtained from the Clinical Research Ethics Committee of the First Affiliated Hospital of Zhejiang University School of Medicine, China (approval number: IIT20210069B-R1). All procedures were performed in accordance with the 1964 Helsinki Declaration and its later amendments or comparable ethical standards. The case group (*n* = 30) included postmenopausal women who had prolapse of POP stage 2 or higher (according to the POP quantification system) and underwent planned vaginal hysterectomy because of POP-related symptoms. The control group (*n* = 30) consisted of women without prolapse who had undergone hysterectomy for other benign diseases. The patients had no oxidative stress-related disorders, including connective tissue disease or coronary heart disease. Patients who had taken estrogen in the past 3 months were excluded from the study. Informed consent was obtained from all individual participants included in the study.

### Tissue Collection and Immunohistochemistry

USL specimens were obtained from tissues extracted by hysterectomy. Samples (approximately 5 mm^3^ in size) were taken from the dorsal part of the cervix, 1 cm from the beginning of the cervix (at the left side), and verified by histological analysis.

Tissue samples were fixed in 10% neutral buffered formalin, embedded in paraffin, and treated with citric acid buffer (pH 6.0) in a microwave for antigen retrieval. Sections were then incubated in 3% hydrogen peroxide solution at room temperature in the dark for 25 min to inhibit endogenous peroxidases, then blocked in 3% bovine serum albumin at room temperature for 30 min. Reactions with primary antibodies against CD44 (dilution 1:100; GB13065), MMP-2 (dilution 1:500; GB11130), MMP-9 (dilution 1:500; GB11132-2), and TGF-β (dilution 1:100; GB13028) (all from Servicebio, Wuhan, China) were performed overnight at 4°C. After incubation with horseradish peroxidase-labeled secondary antibodies (Servicebio) at room temperature for 50 min, immune reactivity was detected with diaminobenzidine (K5007, DAKO, Wuhan, China); color development was observed under a microscope. Nuclei were counterstained with Harris hematoxylin. The percentages of positively stained areas were quantified using ImageJ software (version 1.8.0, National Institutes of Health, Bethesda, MD, USA).

### Statistical Analyses

Data of at least three independent experiments were expressed as mean ± standard deviation. Patient groups were compared using two-sample *t*-tests, Mann–Whitney test, and Fisher’s exact test, separately for means and proportions. Spearman's test was used for the correlation analysis of non-normally distributed continuous variables. Statistical analyses were performed using SPSS (version 23.0; IBM, Armonk, NY, USA) and Prism Version 8.01 (GraphPad Software, La Jolla, CA, USA). Statistical significance was set at *p* < 0.05.

## Results

### Clinical Characteristics of the Study Population

In total, 60 postmenopausal women who had undergone hysterectomy were analyzed: 30 with symptomatic POP (cases) and 30 without POP (controls); their clinical parameters are shown in Table 1. Women with POP were significantly older (*p* < 0.001), had a longer postmenopausal period (*p* < 0.0001), and showed a higher incidence of hypertension (*p* = 0.038) than women without POP. Women in the POP group had a vaginal hysterectomy as the standard surgical treatment for symptomatic POP. In the control group, hysterectomy was performed for uterine leiomyoma (*n* = 20), postmenopausal bleeding (*n* = 2), uterine adenomyosis (*n* = 7), and cervical intraepithelial neoplasia (*n* = 3).

**Table 1:** Clinical characteristics of the study population.

Variable	Case (*n* = 30)	Control (*n* = 30)	*p*-value
Age, years (mean ± SD)	63.9 ± 6.30	55.0 ± 5.60	<0.0001*
Age at menopause, years (mean ± SD)	50.4 ± 2.97	50.0 ± 2.66	0.5536
Years from menopause (mean ± SD)	13.5 ± 7.73	5.03 ± 4.85	<0.0001*
BMI (mean ± SD)	24.1 ± 5.33	24.6 ± 2.98	0.6517
Parity (mean ± SD)	2.07 ± 0.98	1.90 ± 0.66	0.6796
Hypertension, *n* (%)	18 (60)	10 (33.3)	0.038*
Diabetes, *n* (%)	10 (33.3)	3 (10)	0.089

*SD, standard deviation; BMI, body mass index.*

* *Statistically significant.*

### Differential Expression of CD44, TGF-β, MMP-2, and MMP-9 in Women with and Without POP

**Figure 1:**
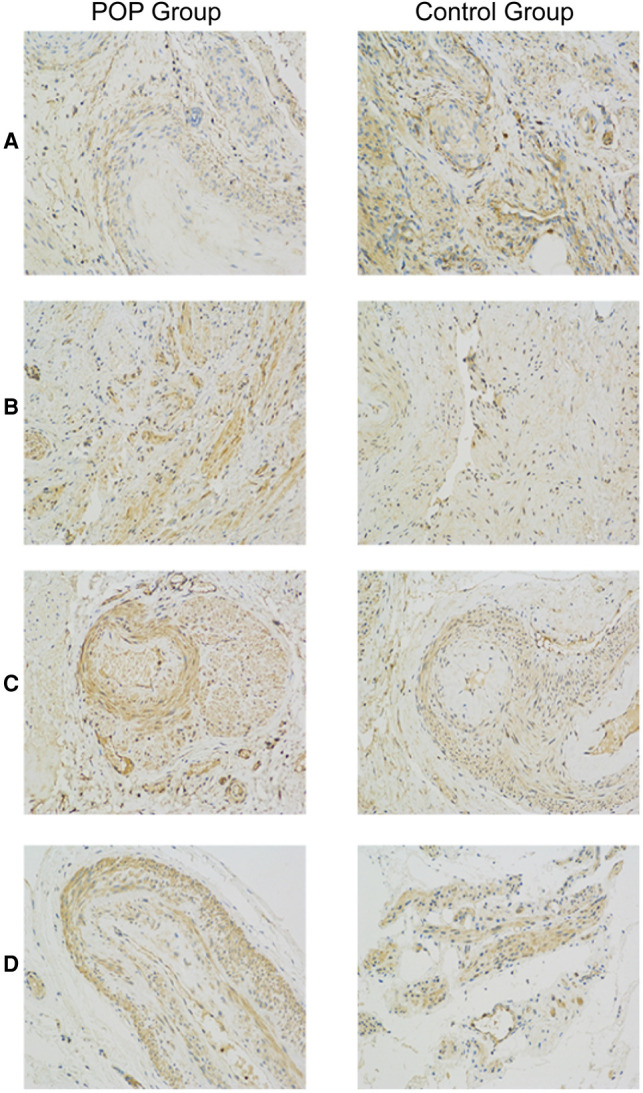
Immunohistochemistry analysis of uterosacral ligaments from women with and without pelvic organ prolapse. Sections of uterosacral ligaments obtained from women who had undergone hysterectomy were stained with antibodies against CD44 (**A**), transforming growth factor-β (**B**), matrix metalloproteinase (MMP)-2 (**C**), and MMP-9 (**D**).

**Figure 2:**
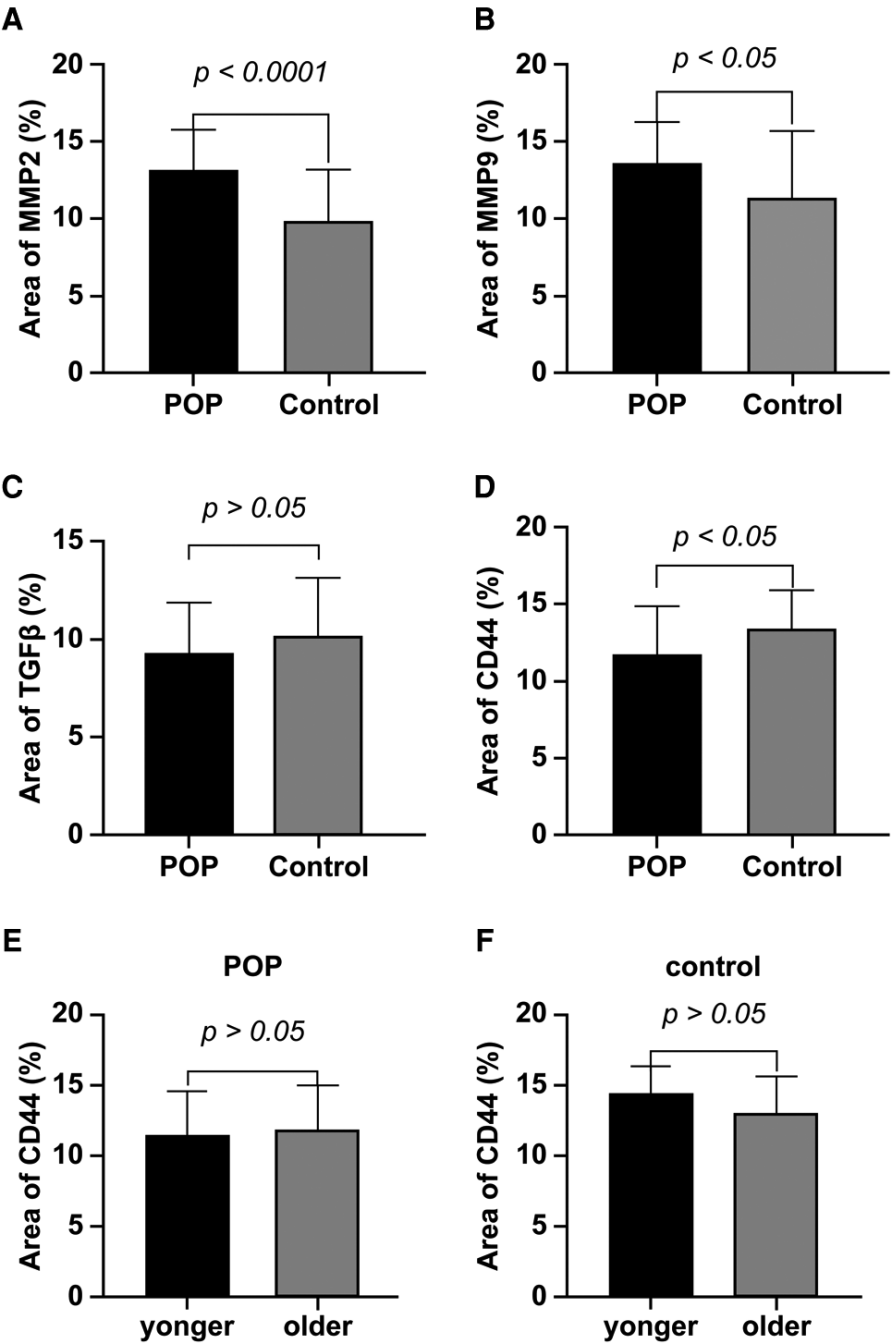
Quantification of matrix metalloproteinase (MMP)-2 (**A**), MMP-9 (**B**), transforming growth factor (TGF)-β (**C**), and CD44 (**D**) expression in uterosacral ligaments. Sections of uterosacral ligaments were stained with antibodies against CD44, TGF-β, MMP-2, and MMP-9, and counterstained with Harris hematoxylin. The percentages of positively stained areas were quantified using ImageJ software and are expressed as means ± standard deviations. POP, pelvic organ prolapse.

Figure 1 shows representative images of USL sections from the POP and control groups after staining with antibodies against CD44, TGF-β, MMP-2, and MMP-9. Quantitative analyses revealed a significant decrease in CD44 expression and an increase in MMP-2 and MMP-9 expression in the POP group compared with those in the control group (Figure. 2). The percentages of USL-positive areas stained with anti-CD44 antibodies were 11.8% in patients with and 13.5% in those without POP (*p* = 0.0263). The CD44 expression levels in younger and older POP patients did not differ significantly from those in younger and older control subjects (11.6% vs. 11.9% and 14.5% vs. 13.1% stained areas in younger and older patients, respectively). The expression levels of MMP-2 and MMP-9 were similar in the POP group (13.2% and 13.7% stained areas, respectively) but significantly higher than those in the control group (9.92% and 11.4%; *p* < 0.0001 and *p* = 0.0155, respectively). However, there was no significant difference in the expression of TGF-β between the POP and control groups (9.34% vs. 10.2%; *p* = 0.2213).

### Risk Factors of Uterine Prolapse

Spearman's correlation analyses were performed to determine the associations of various risk factors with POP. The results indicated that the occurrence of uterine prolapse was positively correlated with age, postmenopausal age, and MMP-2 and MMP-9 expression (*p* < 0.01), and negatively correlated with CD44 expression (*p* < 0.05) (Table 2).

**Table 2:** Correlations between pelvic organ prolapse and risk factors.

Variables	Pelvic organ prolapse	
	Spearman’s correlation	*p*-value
Age	0.582	<0.01*
Age at menopause	0.083	0.531
Years from menopause	0.618	<0.01*
BMI	0.074	0.575
Parity	0.015	0.911
CD44	−0.308	0.017*
MMP-2	0.467	<0.01*
MMP-9	0.346	<0.01*
TGF-β	0.132	0.317

*BMI, body mass index.*

* *Statistically significant.*

## Discussion

In this study, we showed that postmenopausal women with symptomatic POP, and those without POP, differed in the expression of factors involved in the synthesis and turnover of the ECM. Specifically, the levels of CD44 were significantly lower, and those of MMP-2 and MMP-9 were higher in the USLs of the POP group than in the USLs of the control group. Furthermore, CD44 levels were negatively correlated and MMP-2/9 levels positively correlated with prolapse. However, there was no difference in the expression of TGF-β between women with and without POP. These results support our hypothesis that CD44 could be involved in the pathogenesis of POP and are consistent with roles for CD44, MMP-2, and MMP-9 in the mechanisms underlying the balance between the production and degradation of the ECM.

POP is commonly considered as a condition caused by the weakness of pelvic floor muscles and fascia due to childbirth, age, and abnormalities in connective tissue. The ECM is a key component of connective tissue that provides a framework for pelvic support; therefore, it may play a critical role in the occurrence of POP (17). The ECM is dynamically reshaped through quantitative and qualitative changes in cellular and non-cellular components, including enzymes such as collagenase and MMPs that regulate ECM turnover (18). MMPs are closely involved in ECM remodeling in normal and pathological conditions as they can cleave most ECM proteins (19). One of the major functions of MMP-2 is to degrade type IV collagen, elastin, and fibronectin. It is regulated by tissue inhibitor of MMP-2 (TIMP-2), which irreversibly inactivates the enzyme, preventing ECM degradation. MMP-9 is also involved in ECM protein degradation and is implicated in the development of several diseases (20). Previous studies revealed higher expression of MMPs, including MMP-2 and MMP-9, and lower expression of TIMP-2, in USL and vaginal wall biopsies from patients with POP (21, 22). These data, together with our results, suggest that the increased expression of MMP-2 and MMP-9 can accelerate ECM breakdown and promote POP.

TGF-β is considered a critical fibrogenic cytokine that plays a role in myofibroblast differentiation in different tissues (23). The TGF-β/Smad signaling pathway regulates the activity of MMPs and promotes stress-related urinary incontinence after natural birth in animal experiments, whereas TGF-β is downregulated after excessive mechanical strain or oxidative stress (17, 20). However, the role of TGF-β in POP is controversial. For example, a study reports that TGF-β1 expression in patients with POP is significantly reduced (22), whereas another study did not detected any difference in TGF-β1 levels in the USL of women with and without POP (24). In our study, we also did not observe a significant difference in TGF-β expression between the POP and control groups. However, the TGF-β1 pathway is known as a signaling mechanism critical for the cellular response to mechanical stress (17) and, as such, may be related to the pathophysiology of uterine prolapse in postmenopausal women.

To the best of our knowledge, this is the first report on the expression of CD44 in the USL of women with POP. CD44, a cell surface glycoprotein, participates in cell proliferation, adhesion, and migration, as well as in hematopoiesis and lymphocyte activation (25), through binding to its ligands including hyaluronic acid, MMP-9, vascular endothelial growth factor, epidermal growth factor, fibronectin, type I collagen, and osteopontin (11, 26). It has also been shown that fibril-associated collagen XIV can directly interact with CD44 (27). However, the role of CD44 in ECM deposition and fibrogenesis is unclear. On the one hand, some reports indicate that CD44 may promote the synthesis of ECM constituents. For example, it has been reported that CD44-deficient mice show reduced fibroblast infiltration and collagen deposition but increased proliferative activity of fibroblasts (28), and that they also have decreased collagen breakdown and fibrous collagen deposition (29). However, CD44 knockout mice with acute lung injury do not show significant changes in the accumulation of lung interstitial collagen (14). An interesting novel role of CD44 was observed in an *in vitro* study showing that CD44 may be involved in the coordination of cell motility and proliferative responses to ECM stiffening (30). These data suggest that the effects of CD44 on ECM-related processes may depend on the specific environment. Our results, that CD44 expression was significantly associated with POP, may indicate the involvement of CD44 in the pathophysiology of prolapse.

The mechanism of uterine prolapse is complex and not well-understood. Our results implicate CD44, along with MMP-2 and MMP-9, in the development of POP. This is consistent with a previous report on the synergy between CD44, MMP-2, MMP-9, and TGF-β in the physiological mechanism of tissue remodeling (26). Our results can further improve our understanding of the molecular mechanisms underlying POP pathology and contribute to the establishment of novel therapeutic targets in POP.

Our study had some limitations. As it was a case-control investigation, it was not possible to determine the cause-and-effect relationship between POP and protein expression, and we can only refer to the correlation between the occurrence of prolapse and protein expression detected by immunohistochemistry. Furthermore, additional studies involving young patients are needed to establish the role of CD44 in the etiology of POP. Nevertheless, our results suggest an interesting link between CD44 expression and POP in postmenopausal women that has not been reported previously.

In summary, this study provides evidence that CD44 may be a risk factor for uterine prolapse and a novel candidate biomarker of POP. As the prevalence of POP is rapidly increasing with the increase in the aging population, the identification of new biomarkers offers a foundation for the development of improved therapeutic interventions.

## Data Availability

The raw data supporting the conclusions of this article will be made available by the authors, without undue reservation.
